# Social inclusion for children with hearing loss in listening and spoken Language early intervention: an exploratory study

**DOI:** 10.1186/s12887-017-0823-y

**Published:** 2017-03-14

**Authors:** Gabriella Constantinescu-Sharpe, Rebecca L. Phillips, Aleisha Davis, Dimity Dornan, Anthony Hogan

**Affiliations:** 1 School of Allied Health, Australian Catholic University, Melbourne, Australia; 2 http://www.firstvoice.org; 3 Centre for Health Stewardship, ANU Medical School, The Australian National University, Canberra, Australia; 4 The Shepherd Centre, Sydney, Australia; 5 Hear and Say, Brisbane, Australia; 6 School of Health and Rehabilitation Sciences, University of Queensland, Brisbane, Australia; 7 Research School of Psychology, The Australian National University, Canberra, Australia

**Keywords:** Social inclusion, Children, Hearing loss, Listening and spoken language

## Abstract

**Background:**

Social inclusion is a common focus of listening and spoken language (LSL) early intervention for children with hearing loss. This exploratory study compared the social inclusion of young children with hearing loss educated using a listening and spoken language approach with population data.

**Methods:**

A framework for understanding the scope of social inclusion is presented in the Background. This framework guided the use of a shortened, modified version of the Longitudinal Study of Australian Children (LSAC) to measure two of the five facets of social inclusion (‘education’ and ‘interacting with society and fulfilling social goals’). The survey was completed by parents of children with hearing loss aged 4–5 years who were educated using a LSL approach (*n* = 78; 37% who responded). These responses were compared to those obtained for typical hearing children in the LSAC dataset (*n* = 3265).

**Results:**

Analyses revealed that most children with hearing loss had comparable outcomes to those with typical hearing on the ‘education’ and ‘interacting with society and fulfilling social roles’ facets of social inclusion.

**Conclusions:**

These exploratory findings are positive and warrant further investigation across all five facets of the framework to identify which factors influence social inclusion.

## Background

Social inclusion is a common focus of paediatric early intervention, including for service providers addressing the needs of children with hearing loss. Reports have documented that children with hearing loss may experience lower levels of social inclusion than their typical hearing peers and as explored later in the paper, communication skills may be an influential factor [1, 2]. Individuals, the community and government are increasingly recognising the importance of social inclusion due to its widespread impact on areas of the individual’s life including their mental health and economic participation [3, 4]. This has been supported by the inclusion of social inclusion as a desired outcome for children with disabilities in key policy documents, such as the Australian National Disability Agreement (NDA; [5]) and the United Nations Convention on the Rights of Persons with Disabilities [6].

### Conceptual Underpinnings and Measurement of Social Inclusion

Despite the acknowledgment of social inclusion and its positive benefits, a common understanding of its conceptual underpinnings and approaches to measurement has not been reached [7, 8]. Social inclusion is measured using varying methods across the different fields of economics, education and health, which may contribute to this inconsistency. Discussion in these fields centre on two perspectives; a policy or a clinical perspective (specifically for individuals with disabilities). From a policy perspective, indicators of economic self-sufficiency and education are mainly used to measure social inclusion [9, 10]. In the clinical context, literature on the social inclusion of children with disabilities primarily focuses on social interactions, school participation and the way that children with disabilities are included in these contexts. Much of the literature examining the social inclusion of children with disabilities focuses on the type of school that the child attends. This views social inclusion as a state (e.g. that all students should attend mainstream schools) [11]. However, social inclusion can also be seen as a process by which participation is increased, or as a means to an end (e.g. academic outcomes are improved in mainstream schools), or more broadly as a value system concerned with human rights and based on citizenship and community membership [11, 12]. Through the lens of a value system, social inclusion is viewed as broader than the way that a child is accepted and included within an education setting by also incorporating community and all aspects of the child’s life [11, 12]. It is implied within a value system that the classroom and community need to change to accommodate the child with different needs, and it is not simply about the child being present in those environments [2, 13]. This is highlighted by Hyde who explains, “included students are mainstreamed but not all mainstreamed students may be included.” ([2], p93). For example, a child may be in an inclusive school or community environment but may not feel included if they are bullied by peers who do not understand how to support their needs [14–17]. Therefore, experiencing a sense of belonging is increasingly seen to be a key element of social inclusion [18].

### Framework for Social Inclusion

The policy and clinical perspectives of social inclusion have been captured within a framework depicting five facets (perspectives) of social inclusion: economic participation, health and access to services, education, personal independence and determination, and interacting with society and fulfilling social roles [19]. The five facets of social inclusion demonstrate that there is more to social inclusion than economic participation or participation in the school environment. They also demonstrate the complexity involved in measuring social inclusion. In this paper, social inclusion will be defined as the purposeful connection of the individual with their social setting rather than purely being present in the setting.

### Social Inclusion of Children with Hearing Loss

As previously mentioned, it is reported that children with hearing loss may experience lower levels of social inclusion than their typical hearing peers and communication skills may be an influential factor [1, 2]. The focus of these studies on adolescents [1] and the narrow view of social inclusion in only education settings [2] limits the applicability of their findings to understanding the social inclusion of children with hearing loss across the breadth of everyday life as outlined in the above framework.

There are studies, although not explicitly focused on social inclusion, that do provide some insight into the domains listed in the framework, particularly in relation to the facet of ‘interacting with society and fulfilling social roles’. For example, there is evidence to suggest that children with hearing loss under 10 years of age have difficulties with: attention [20]; behaviour [20–22]; communication [20]; and relationships [23]. This literature indicates that children with hearing loss experience restricted levels of social inclusion on these domains of the ‘interacting with society and fulfilling social roles’ facet. In comparison, a study by Percy-Smith, Cayé-Thomasen, Gudman, Jensen & Thomsen [24] took a slightly broader view, investigating self-esteem and social well-being, which also fit within the proposed new facet of ‘interacting with society and fulfilling social roles’. This study included 164 children with cochlear implants (educating using listening and spoken language or sign language) and 2169 typical hearing children aged 2–17 years. The authors found only minor differences between the two groups, with children with cochlear implants scoring the same, if not better than the typical hearing children on aspects of self-esteem and social well-being [24].

A more recent study by Warner-Czyz and colleagues also reported similar findings for self-esteem, where the 50 children and adolescents with hearing loss in the study had significantly higher ratings of self-esteem to hearing peers [25]. The children with hearing loss were aged eight to 18 years, aided using hearing aids or cochlear implants and had received listening and spoken language early intervention. Additional factors to influence self-esteem in the study included personal behaviours such as attention, affiliation, temperament and mood. Communication skills (as rated by the participants), participant age, age of fitting of hearing aids or cochlear implants, duration of use and social engagement (e.g. participation in activities and friendships) were not found to be linked to self-esteem [25].

In a previous study, we investigated the impact of spoken language skills on social inclusion for 95 children with hearing loss aged 5 years [19]. Using the facets of ‘education’ and ‘interacting with society and fulfilling social roles’, it was found that speech skills and vocabulary impacted social inclusion. When looking at the facet of ‘education’, speech skills were predictive of conduct skills such as the children’s ability to regulate behaviour, and use of social resources such as computers. For the latter, it was suggested that children with better speech skills spent more time using technology rather than needing more structured time with parents to work on their speech. Vocabulary skills also predicted parent satisfaction with teacher support, where higher skills reflected higher satisfaction. On the facet of ‘interacting with society and fulfilling social roles’, vocabulary skills also predicted whether the children with hearing loss were invited to social activities such as birthday parties. Better skills were likely to reflect more meaningful interactions with peers and being invited to social events [19].

### Study Aims

To contribute to the existing knowledge, this study aimed to investigate the social inclusion of children aged 4–5 years who are educated using listening and spoken language early intervention (a cohort for which literature on this area is particularly limited) and compare these outcomes to those of their typical hearing peers. The study focused on the two facets of social inclusion using the new framework which were most relevant at the clinical level for this age group and closely related to the goals of listening and spoken language early intervention:

- education: encompassing education participation and education skills, and
- interacting with society and fulfilling social roles: encompassing acceptance, perceived social inclusion, social networks, role functioning and engagement, behaviour, social participation, political and civic participation, and social resources [19].

Although the facet ‘personal independence and self-determination’ is relevant at the clinical level, its applicability is limited for children under 5 years where caregivers largely influence choice and independence and not included here. The ‘economic participation’ and ‘health and access to services’ facets were not included because they are more related to a policy rather than clinical perspective. The knowledge from this study will further contribute to the evidence-base in relation to social inclusion outcomes, which is currently lacking for children with hearing loss, particularly young children.

## Method

A modified version of the Longitudinal Study of Australian Children (LSAC) was used to compare the social inclusion of young children with hearing loss, against data from the LSAC. The LSAC is broadly representative of the Australian population, following two cohorts of children every 2 years (infants aged 0–1 years and children aged 4–5 years) to investigate experiences of: children within their families and communities; their health; child care experiences; and their early years of education [26, 27]. The focus of the LSAC is broader than social inclusion but includes items that provide insight into the domain and indicators representing the five facets of social inclusion. The LSAC survey design was rigorously developed, including pre-testing and validation of the questions [27, 28]. Items were included in the LSAC if they met the following criteria: established reliability and validity; acceptability to respondents; ability to measure central constructs; comparability with other international or national datasets; and lack of redundancy [27].

Ethics approval for this research was gained from the Behavioural and Social Sciences Ethical Review Committee of the University of Queensland, Australia, as well as project approval from First Voice member centres. The authors obtained a license to use the LSAC data from the Australian Government Department of Social Services.

### Children with Hearing Loss

#### Participants

Primary caregivers were eligible to complete the survey about their child’s social inclusion if their child: (1) was in the age range of 4–5 years at the time of the survey; (2) had been identified with a permanent bilateral hearing loss; (3) was optimally aided (hearing aids and/or cochlear implants); (4) was enrolled in listening and spoken language early intervention for at least 6 months; and (5) had no physical or cognitive difficulties that their medical and clinical team judged as impacting on learning.

Of the 212 children and primary caregivers eligible and contacted for study inclusion, 78 primary caregivers completed the social inclusion survey. This 37% response rate is consistent with the typical rates of less than 50% that are reported for cross-sectional surveys, as well as rates of 39% for online surveys [29, 30]. It is likely that a number of factors may have influenced completion rate such as primary caregivers being too busy and already juggling other demands of looking after their young children and attending early intervention; not being interested in the project; or not seeing the direct relevance or benefit of participating to their child. The demographics of the children with hearing loss included in the study are presented in Table 1. Spoken language outcomes for the group using standardised assessments showed performance within the average range (Standard Scores; SS between 85 and 115) on measures of vocabulary (*M* = SS 95.24; *SD* = SS 19.81) using the Peabody Picture Vocabulary Test-4 [31]; language (*M* = SS 93.07; *SD* = SS 19.82) using the Preschool Language Scale-4 [32], the Clinical Evaluation of Language Fundamentals-Preschool [33], or the Clinical Evaluation of Language Fundamentals-4 [34]; and speech (*M* = SS 93.26; *SD* = SS 16.42) using the Goldman-Fristoe Test of Articulation-2 [35].

**Table 1:** Demographics of children with hearing loss

Characteristics	All participants (*N* = 78)
Severity of hearing loss	
Mild	*n* = 8 (10.3%)
Moderate	*n* = 19 (24.4%)
Moderately severe	*n* = 18 (23.2%)
Severe	*n* = 5 (6.4%)
Profound	*n* = 28 (35.9%)
Aetiology of hearing loss	
Unknown	*n* = 38 (48.7%)
Acquired	*n* = 1 (1.3%)
Congenital non-genetic	*n* = 11 (14.1%)
Genetic non-syndromic	*n* = 19 (24.4%)
Syndromic	*n* = 9 (11.5%)
Age hearing loss identified	*M* = 0.84 years (*SD* = 1.11 years); Range = 0.00–3.91 years
Amplification type	
Hearing aids—bone anchored	*n* = 1 (1.3%); age at fitting = 0.5 years
Hearing aids—bilateral	*n* = 38 (48.7%); age at fitting of first hearing aid *M =* 1.09 years (*SD =* 1.3 years); Range = 0.08–4.00 years
	age at fitting of bilateral hearing aid *M* = 1.13 (*SD =*1.24 years); Range = 0.08–4.00 years
Bimodal aiding (i.e. combined cochlear implant and hearing aid users)	*n* = 14 (17.9%); age at fitting of hearing aids *M* = 1.32 years (*SD* = 1.19 years); Range = 0.16–2.83 years
	age at implant *M* = 2.50 years, (*SD* = 1.17 years); Range = 1.16–3.83 years
Bilateral cochlear implant users	*n* = 25 (32.1%)
	age at first implant *M* = 1.45 years (*SD* = 1.16 years); Range = 0.41–4.66 years
	age at bilateral implant *M* = 1.67 years (*SD* = 1.13 years); Range = 0.41–4.66 years
Age enrolled in listening and spoken language early intervention programs	*M* = 1.25 years (*SD* = 1.24 years); Range = 0.00–3.91 years
Length of time in early intervention program at time of study	*M* = 3.67 years (*SD* = 0.98 years); Range = 1.08–5.16 years

The primary caregivers were mainly the mothers. Within the home environment, English only was predominantly spoken (87%), while the remainder of families spoke English and another language such as Chinese and Māori. For the primary caregivers, the highest level of education included university or equivalent (71%); year 11 or 12 or equivalent (16%; the last 2 years of high/secondary school); trade or equivalent which included technical education outside of high/secondary school (5%); and year 10 or below (8%).

#### Procedure

Participants were recruited through the five First Voice member centres in Australia and an affiliated centre in New Zealand. Established in 2010, First Voice is the national voice for member centers in Australia and an affiliated center in New Zealand. The primary focus of First Voice is the provision of listening and spoken language early intervention for children with hearing loss.

Eligible children were identified from the First Voice central database. Primary caregivers were contacted for participation via mail or in-person during the child’s therapy session. At this time, primary caregivers were provided with an information sheet and consent form for participation in the survey and sharing of their child’s demographic information. Once written consent was obtained, primary caregivers were emailed a link to the online survey or provided with a paper version, as per individual preference.

#### Survey

A modified version of the LSAC was used to collect data on the two facets of social inclusion: ‘education’ (measurement domains of education participation and education and skills) and ‘interacting with society and fulfilling social roles’ (measurement domains of acceptance, social networks, role functioning and engagement, behaviour, social participation and social resources). The social inclusion survey contained 62 items and took approximately 30 min to complete.

The survey primarily included questions from the LSAC survey but also included some purpose designed items to capture aspects of social inclusion not measured by the LSAC. Responses to these purpose designed items are not reported in this article, which focuses on comparing the outcomes of children attending First Voice centres to the LSAC dataset. Survey items that were used in the analysis for this study are listed in Tables 2, 3, 4, 5 and 6.

**Table 2:** Comparing the education participation of children with and without hearing loss

			Hearing loss			
Item	HL *n* (%)	No HL *n* (%)	OR (95% CI)	Wald	*P*	*SE*
Number of days, on average, attended an education program^a^						
4 or more days/week (reference: 3 or fewer, or irregular)	30 (38.5)	1017 (31.2)	0.87 (0.52–1.43)	0.32	.574	.256
Number of hours, on average, spent in education program each week^b^						
7 or more (reference: 0–6 or irregular)	61 (81.3)	2985 (96.1)	0.13 (0.07–0.26)	32.31	<.001*	.358
How well child’s classroom teacher, centre or preschool does at letting the primary caregiver know about their child’s progress in the program or class^c^						
Very well or well (reference: just okay, not done at all or don’t know)	59 (77.6)	2445 (78.9)	0.93 (0.52–1.65)	0.06	.802	.294
How well child’s classroom teacher, centre or preschool does at helping the primary caregiver understand what children at their child’s age are like^c^						
Very well or well (reference: just okay, not done at all or don’t know)	52 (70.3)	2110 (68.1)	1.02 (0.60–1.73)	0.01	.934	.269
How well child’s classroom teacher, centre or preschool does at making the primary caregiver aware of chances to be involved and take part in the centre or school^c^						
Very well or well (reference: just okay, not done at all or don’t know)	61 (82.4)	2529 (81.6)	0.84 (0.44–1.60)	0.30	.585	.330
How well child’s classroom teacher, centre or preschool does at giving the primary caregiver information and advice about how to help their child at home^c^						
Very well or well (reference: just okay, not done at all or don’t know)	45 (61.6)	1928 (59.4)	0.89 (0.54–1.46)	0.23	.635	.252
Satisfaction with educational program^d^						
Very satisfied or satisfied (reference: not satisfied)	72 (96.0)	2885 (93.1)	1.51 (0.47–4.89)	0.47	.491	.599

*n* (*%*) the number and percentage of participants who provided this response following the conversion of the question to a dichotomous variable; *HL* hearing loss; *OR* odds ratio; *CI* confidence interval; * = statistically significant after Bonferroni adjustment (adjusted alpha = 0.05/7 = 0.007. Analyses were adjusted for the child’s age and the primary caregiver’s level of education

^a^Response options on survey: 1; 2; 3; 4; 5; 6; irregular days

^b^Response options on survey: 0–3; 4–6; 7–9; 10+; irregular hours

^c^Response options on survey: very well; well; just okay; not done at all; don’t know

^d^Response options on survey: very satisfied; satisfied; neither satisfied nor dissatisfied; dissatisfied; very dissatisfied

**Table 3:** Comparing the education skills of children with and without hearing loss

Item			Hearing loss			
	HL *n* (%)	No HL *n* (%)	OR (95% CI)	Wald	*P*	*SE*
Number of children’s books child has at home, including library books^a^						
More than 21 (reference: 20 or fewer)	75 (96.1)	2936 (90.0)	3.38 (0.81–14.13)	2.78	.096	.730
Child is able to read simple words like ‘dog’ or ‘cat’^b^	24 (30.8)	1123 (34.5)	0.53 (0.31–0.90)	5.61	.018*	.269
Child is able to read complex words like ‘table’ and ‘orange’^b^	6 (7.7)	254 (7.8)	0.70 (0.29–1.72)	0.60	.440	.456

*n* (*%*) the number and percentage of participants who provided this response following the conversion of the question to a dichotomous variable; *HL* hearing loss; *OR* odds ratio; *CI* confidence interval; * = statistically significant after Bonferroni adjustment (adjusted alpha = 0.05/3 = 0.02. Analyses were adjusted for the child’s age and the primary caregiver’s level of education

^a^Response options on survey: none; less than 10; 10–20; 21–30; more than 30

^b^Response options on survey: yes; no

**Table 4:** Comparing role functioning and engagement between children with and without hearing loss

Item				Hearing loss			
		HL *n* (%)	No HL *n* (%)	OR (95% CI)	Wald	*P*	*SE*
Number of days in the past week that someone in the family has done the following with the child							
	Read to child from a book^a^ 3 or more days (reference: 2 or fewer days)	74 (94.9)	2508 (76.9)	8.31 (2.03–34.06)	8.65	.003*	.720
	Told a story, not from a book^a^ 3 or more days (reference: 2 or fewer days)	27 (35.1)	918 (28.2)	1.59 (0.98–2.60)	3.48	.062	.250
	Drawn pictures or did other art or craft^a^ 3 or more days (reference: 2 or fewer days)	53 (67.9)	1871 (57.4)	1.65 (1.00–2.73)	3.82	.051	.257
	Played music, sang songs, danced or did other musical activities^a^ 3 or more days (reference: 2 or fewer days)	52 (66.7)	1922 (59.0)	1.54 (0.94–2.53)	2.89	.089	.254
	Played with toys or games indoors, like board or card games^a^ 3 or more days (reference: 2 or fewer days)	52 (67.6)	1687 (51.7)	2.20 (1.33–3.64)	9.37	.002*	.257
	Involved child in everyday activities at home^a^ 3 or more days (reference: 2 or fewer days)	50 (64.1)	2219 (68.1)	0.98 (0.60–1.61)	0.01	.944	.251
	Played a game outdoors or exercised together like walking, swimming, cycling^a^ 3 or more days (reference: 2 or fewer days)	46 (59.0)	1971 (60.5)	1.05 (0.65–1.69)	0.04	.842	.243
When has a choice about how to spend free time, child usually chooses active pastimes or just as likely to choose active or inactive pastimes^b^ (reference: usually chooses inactive pastimes)		53 (68.0)	2474 (75.8)	0.77 (0.46–1.29)	1.01	.316	.262

*n* (*%*) the number and percentage of participants who provided this response following the conversion of the question to a dichotomous variable; *HL* hearing loss; *OR* odds ratio; *CI* confidence interval; * = statistically significant after Bonferroni adjustment (adjusted alpha = 0.05/8 = 0.006. Analyses were adjusted for the child’s age and the primary caregiver’s level of education

^a^Response options on survey: none; 1 or 2 days; 3–5 days; every day (6–7 days)

^b^Response options on survey: usually chooses inactive pastimes like TV, computer, drawing or reading; just as likely to choose active as inactive pastimes; usually chooses active pastimes like bike riding, dancing, games or sports; don’t know

**Table 5:** Comparing social participation between children with and without hearing loss

Item				Hearing loss			
		HL *n* (%)	No HL *n* (%)	OR (95% CI)	Wald	*P*	*SE*
Activities completed with a family member in the past month							
	Gone to a movie^a^	42 (53.8)	1256 (38.5)	1.68 (1.06–2.69)	4.77	.029	.238
	Gone to a playground/swimming pool^a^	75 (96.2)	3056 (93.6)	1.98 (0.46–8.44)	0.85	.356	.740
	Gone to a sporting event where child was not a player^a^	36 (46.2)	1387 (42.5)	1.17 (0.73–1.87)	0.44	.507	.238
	Gone to a live performance for children^a^	2456 (75.2)	806 (24.7)	1.62 (1.00–2.63)	3.81	.051	.248
	Attended a school cultural or community event^a^	57 (73.1)	1705 (52.2)	1.93 (1.15–3.23)	6.21	.013	.263
	Attended a religious service^a^	23 (29.5)	1127 (34.5)	0.59 (0.35–0.99)	3.93	.047	.268
	Visited a library^a^	49 (62.8)	1710 (52.4)	1.26 (0.78–2.03)	0.87	.350	.245
	Visited a museum or art gallery^a^	18 (23.1)	543 (16.6)	1.34 (0.77–2.32)	1.09	.297	.280
Special/extra cost activities completed in the last 6 months							
	Swimming^a^	37 (47.4)	1499 (45.9)	0.89 (0.56–1.42)	0.23	.629	.238
	Gymnastics/kinder gym^a^	10 (12.8)	327 (10.0)	1.17 (0.59–2.32)	0.19	.665	.352
	Team sports (e.g. athletics, football)^a^	12 (15.4)	316 (9.7)	1.44 (0.76–2.72)	1.24	.266	.326
	Musical instruments or singing^a^	3 (3.8)	168 (5.1)	0.61 (0.19–1.98)	0.67	.414	.599
	Ballet or other dance^a^	13 (16.7)	481 (14.7)	0.94 (0.50–1.77)	0.04	.851	.322
	Children’s religious group (e.g. Sunday school)^a^	6 (7.7)	425 (13.0)	0.48 (0.19–1.20)	2.46	.117	.469
	Other^a^	15 (19.2)	166 (5.1)	4.24 (2.30–7.82)	21.43	<.001*	.312

*n* (*%*) the number and percentage of participants who provided this response; *HL* hearing loss; *OR* odds ratio; *CI* confidence interval; * = statistically significant after Bonferroni adjustment (adjusted alpha = 0.05/15 = 0.003. Analyses were adjusted for the child’s age and the primary caregiver’s level of education

^a^Response options on survey: yes; no

**Table 6:** Comparing summary scores on the SDQ scales between children with and without hearing loss

			Hearing loss			
Item	HL *n* (%)	No HL *n* (%)	OR (95% CI)	Wald	*P*	*SE*
Normal score on SDQ hyperactivity scale	67 (85.9)	2751 (84.4)	0.96 (0.50–1.85)	0.02	.903	.335
Normal score on SDQ emotional symptoms scale	71 (91.0)	2885 (88.6)	1.19 (0.53–2.68)	0.17	.677	.415
Normal score on SDQ conduct problems scale	61 (78.2)	1909 (58.6)	2.32 (1.32–4.07)	8.59	.003*	.287
Normal score on SDQ peer problems scale	64 (82.1)	2496 (76.6)	1.28 (0.69–2.37)	0.63	.427	.313
Normal score on SDQ prosocial scale	66 (84.6)	2891 (88.7)	0.74 (0.38–1.45)	0.76	.384	.340
Normal score on SDQ total difficulties scale	68 (87.2)	2718 (83.4)	1.11 (0.56–2.20)	0.08	.773	.351

*HL* hearing loss; *OR* odds ratio; *CI* confidence interval; * = statistically significant after Bonferroni adjustment (adjusted alpha = 0.05/6 = 0.008. Analyses were adjusted for the child’s age and the primary caregiver’s level of education

An expert panel of four researchers and clinicians identified items in the LSAC that addressed the indicators of the ‘education’ and ‘interacting with society and fulfilling social roles’ facets in the framework, thus allowing comparison to this data set. These items were then incorporated in the study survey. For example, to measure the domain of ‘behaviour’ within the ‘interacting with society and fulfilling social roles’ facet, the panel selected the Strength and Difficulties Questionnaire (SDQ). The SDQ is a 25 item survey that provides summary scores for 5 scales: emotional symptoms, conduct problems, hyperactivity/inattention, peer relationship problems and prosocial behaviour [36]. The SDQ collected information about the behaviours displayed by the child and provided insight into what the quality of the interactions may be for them. The psychometric properties of the SDQ have been assessed in 4–9 year old Australian children (*n* = 1359) [37]. Internal reliability across the subscales was reported to be moderate to strong, test-retest reliability was stable over 12 months. The panel proceeded to work through each domain within the two facets to select the items that addressed the indicators outlined by Constantinescu et al. [19].

Initially, a more comprehensive survey was developed to address all five facets of social inclusion. However, feedback from pilot testing (*n* = 10 primary caregivers) suggested this to be too time intensive and likely to reduce participation. Bearing this in mind, the expert panel narrowed down the survey to the two facets of social inclusion (education; and interacting with society and fulfilling social roles) most relevant at the clinical level.

### Children without Hearing Loss

Cross-sectional data for typical hearing children from Wave 1 (2004) of the existing LSAC dataset were used as a comparison group. Data were included in this study for children who were: (1) aged 4–5 years; and (2) were not reported to have the following health conditions: hearing problems, vision problems, Attention Deficit Disorder, eczema, diarrhoea or colitis, frequent headaches, ear infections, other infections, food or other allergies, other illnesses and other physical disabilities. Applying these criteria to the data resulted in 3265 participants.

Children included in the LSAC cohorts are broadly representative of the Australian population [26]. Children from single-parent, non-English speaking families and those living in rental properties were slightly underrepresented, while children with more highly educated parents were slightly over-represented (by around 10 percentage points). LSAC data were collected from mothers, fathers, carers or teachers and direct observations of the children. However, primary caregivers (98% mothers) were the respondent for the items analysed in this paper.

### Analysis

To detect differences between the children with hearing loss and typical hearing children on the demographic variables, scale data were analysed using independent-samples t-tests and Levene’s test for equality of variances, and categorical data using Pearson chi-square analyses (with Yates continuity correction when both categorical variables only had two categories). Results were considered significant at *p* < .050. Logistic regression was used to compare the two groups on the two facets of social inclusion (‘education’ and ‘interacting with society and fulfilling social roles’), adjusting for the child’s age and primary caregiver’s highest level of education (both of which were found to be higher for the group of children with hearing loss). For the logistic regression, categorical items from the social inclusion survey were recoded as dichotomous variables (see Tables 2, 3, 4, 5 and 6). The adjusted odds ratios and 95% confidence intervals of these comparisons are reported in each table. The odds ratio indicates how likely it is that an individual who displayed a certain characteristic belongs to the group of children with hearing loss rather than the group of children with typical hearing. For example, the first item in Table 4 indicates that the parents of children with hearing loss are 8.31 times more likely than the parent of children with typical hearing to read to their child from a book 3 or more days a week. The *p* value (.003) indicates that this finding is statistically significant following Bonferroni adjustment. Bonferroni adjustments were used to reduce the risk of Type 1 error due to the multiple comparisons included in these analyses. The Bonferroni corrected significance level is listed below each table.

## Results

### Comparison of Demographics Between Groups

When comparing the two groups for gender, no differences were found (55.1% males for the children with hearing loss; 49.4% males for the typical hearing children). At the time of survey, the children with hearing loss were significantly older (*t* =−7.40; *p* < .001), with a mean age of 4.93 years (*SD* = 0.54 years) compared to a mean age of 4.74 years (*SD* = 0.22 years) for the children with typical hearing.

The type of education program attended by the children with hearing loss was primarily (39.7%) pre-year 1 program in a school. This is seen as the first year of formal schooling where typically the children would be 5 years of age or turning 5 during that year. The next highest attendance (23.0%) was pre-school, which is the year prior to formal schooling where typically the children would be 4 years of age or turning 4 during that year. The remaining children (17.9%) attended day/child care, which is the year prior to pre-school where typically the children would be 3 years of age or younger during that year. These results were somewhat different to the percentage of children with typical hearing who primarily attended a pre-year 1 program (16.8%) and pre-school program (55.7%), while similar for day/child care (18.4%) attendance. These differences may be reflective of the group with hearing loss being significantly older. For the type of care apart from school or pre-school, the hearing loss group was significantly more likely than the typical hearing group to also receive care from a day care where the child has a pre-school program (χ^2^ = 11.41; *p* = .001); gym, leisure or community care (χ^2^ = 6.20; *p* = .013); grandparent (χ^2^ = 23.66; *p* < .001); or another relative (χ^2^ = 10.99; *p* = .001).

There was a significant difference between the groups for primary caregiver’s level of education (χ^2^ = 21.62; *p* < .001), with the hearing loss group more likely to have completed year 11–12 or equivalent (96.0% versus 71.7% for the typical hearing group). The typical hearing group was more likely than the hearing loss group to have only completed year 10 or below or equivalent (28.2% versus 4.0% for the hearing loss group), or to never have attended school (0.1% versus 0.0% for the hearing loss group). Due to the significant differences between the two groups for the child’s age and primary caregiver’s highest level of education, adjustments were made during analysis to account for this.

### Comparison of Social Inclusion Between Groups

This paper focused on comparing the social inclusion of children aged 4–5 years who are educated using listening and spoken language early intervention to the outcomes of their typical hearing peers. The facets of social inclusion investigated in this study were ‘education’ and ‘interacting with society and fulfilling social roles’. The results related to these two facets are presented in the next sections.

#### Education

Within the facet of ‘education’, the areas of education participation and education skills were examined. Table 2 compares the education participation of the two groups of children. Children with hearing loss were 87% less likely (odds ratio = 0.13) to attend an education program 7 h or more per week (*p* < .001). Significant differences were not found between the groups for the caregiver’s satisfaction with the education program or communication with the classroom teacher. Table 3 reports on the education skills of the children in both groups. The only significant difference was that children with hearing loss were 47% less likely (odds ration = 0.53) to be able to read simple words like ‘dog’ or ‘cat’ (*p* = .018). However, there was not a significant difference between the two groups on their ability to read complex words.

#### Interacting with Society and Fulfilling Social Roles

Within the facet of ‘interacting with society and fulfilling social roles’, the areas of role functioning and engagement, social participation and behaviour were examined. Table 4 shows that as part of role functioning and engagement, the children with hearing loss were significantly more often involved in two leisure activities at home than the typical hearing children, i.e. they were: 8.31 times more likely to be read to by their parents (*p* = .003); and 2.20 times more likely to play indoor games (*p* = .002).

In looking at social participation, Table 5 shows that the children with hearing loss were 4.24 times more likely than the typical hearing group to have participated in other special/extra cost activities (*p* < .001). No other significant differences were reported between the two groups when comparing the types of activities completed outside of the home.

Table 6 presents the overall scale scores for the SDQ, which provide insight into behaviour. Only one area of significant difference (*p* = .003) was found between the two groups and this was on the conduct problems scale. The children with hearing loss were 2.32 times more likely than the typical hearing children to be rated as ‘normal’ on this scale and less likely to be rated as ‘abnormal’.

## Discussion

This study compared the social inclusion of young children with hearing loss educated using listening and spoken language early intervention to typical hearing children, using two facets of social inclusion (education; and interacting with society and fulfilling social roles). This is the second study by the authors to apply the conceptual framework for evaluating social inclusion presented in the Background in order to consider the social inclusion of this cohort. The study findings showed that most of the children with hearing loss had comparable social inclusion to the typical hearing children on the two facets of ‘education’ and ‘interacting with society and fulfilling social roles’.

### The Education Facet of Social Inclusion

On the ‘education’ facet of social inclusion, similarities were generally found between the education skills of the children with hearing loss and those with typical hearing. The interesting finding here is that although the children with hearing loss were significantly less likely to be able to read simple words than the typical hearing children, they were equally as likely to read complex words. These contrasting outcomes have highlighted the need for further research using in-depth questions or assessments relating to literacy to better determine any possible trends in performance for the groups and the extent to which there may be differences between them. Dornan and colleagues [38] did touch on some similarities in reading performance between seven children with hearing loss aged 8 years and seven typical hearing children in their study, where both groups demonstrated skills within the average range. However, further research is needed to add to the current limited knowledge in the area of literacy development for children educated using listening and spoken language early intervention.

Overall, the outcomes for language, vocabulary and speech assessments at the time of the study highlighted, on average, the age-appropriate spoken language development of the children with hearing loss. When comparing education participation, the differences identified may indicate that the children with hearing loss were spending more time within the home environment with their primary caregivers, grandparents or other relatives as noted in the results. The children with hearing loss were more likely to spend less time (specifically 6 h or less or irregularly) in an education program than typical hearing children. This finding is in keeping with the overall philosophy guiding listening and spoken language early intervention where the primary caregiver is seen as the facilitator of the child’s language enriched environment [39]. Meaningful time spent together is seen as optimizing the listening and spoken language development of the child.

### The Interacting with Society and Fulfilling Social Roles Facet of Social Inclusion

When looking at this facet of social inclusion, the children with hearing loss were involved to a similar level as typical hearing children in activities relating to the domains of social participation and role functioning and engagement. These findings are similar to the Percy-Smith et al. [24] study of self-esteem and social well-being in children aged 2–17 years with cochlear implants. The outcomes in the present study may be related to the primary caregiver’s level of understanding and confidence to engage children in activities both at home and in the community to facilitate the child’s social development. It would be interesting in further studies to look at factors (e.g. parental factors or type of early intervention received) that may drive this comparable finding. This will help to identify areas where primary caregivers and their children can be supported to achieve optimal social inclusion outcomes.

As well as knowing about the child’s involvement in a range of activities, it is important to also know about the quality of their interactions so that a true representation of their social inclusion in these activities can be gained. In the present study it was promising to see that on the SDQ overall scale scores that reflect quality of interactions, most of the children with hearing loss had comparable behaviours, emotions and relationships to children with typical hearing, and further, were more likely to have appropriate conduct skills. These findings are similar to those noted by Wong and colleagues [40] in their population based study where on average, the children with hearing loss at 5 years had SDQ scores on all domains within the average range. The authors noted that factors such as no additional disabilities and higher language, functional communication and non-verbal cognitive skills predicted psychosocial functioning. Studies that consider additional factors noted clinically such as the presence and impact of bullying are also needed here. Together, the findings would assist in supporting children when transitioning to the education setting and encountering new experiences in which social inclusion occurs.

### Implications for Practice

The study reported in this paper applied two of the facets of social inclusion to measure the social inclusion of children with hearing loss in listening and spoken language early intervention. This is the second study to investigate the social inclusion of this cohort of children. It is promising that most of the children with hearing loss had comparable levels of social inclusion to typical hearing children. This positive finding is a foundation for future studies to explore specific factors that contribute to these comparable levels of social inclusion. This knowledge will assist primary caregivers and early intervention providers to support children with hearing loss to achieve optimal social inclusion outcomes.

### Limitations

The study findings provide preliminary insights into the social inclusion of this cohort on the facets of ‘education’ and ‘interacting with society and fulfilling social roles’. It is acknowledged that the clinical nature of this research may limit the generalizability of the study findings. For example, this was a relatively small group comparison to a population based study, and participation was sought from an existing early intervention cohort where self selection of the primary caregivers to the type of early intervention needs to be recognised.

### Future Directions

In future, by looking at social inclusion as part of a population based study of children with hearing loss such as the Longitudinal Outcomes of Children with Hearing Impairment study [41] may provide broader insights across the types of early intervention offered in Australia. Further population based studies are also needed worldwide to look at similarities and differences across regions. Clinically, these studies will give service providers the tools to identify areas where lower social inclusion is experienced for children with hearing loss, and the domains that could be integrated into goal setting to address their individual needs.

Additionally, a greater representation of social inclusion would be gained by including children with unilateral hearing losses and those with more complex needs in evaluations (i.e. those with physical and cognitive difficulties impacting on learning). These groups also represent the children with hearing loss seen clinically in Australia and overseas and it would be important to explore their social inclusion. The findings from these studies would help to identify clinical areas for supporting individual children in their social inclusion. Such an investigation was outside the scope of the present study.

Further longitudinal large-scale studies involving greater participant numbers (only 37% of primary caregivers responded to the current survey); all five facets of social inclusion; and with child rather than parental report (where applicable) are needed to add to the current knowledge from this study of the impact of listening and spoken language early intervention on the social inclusion of children with hearing loss. Such studies will provide a more comprehensive picture of the social inclusion across all five facets for children with hearing loss in relation to typical hearing children and from the perspective of the children themselves (where applicable at older ages).

## Conclusions

In summary, this study investigated social inclusion by using two facets of a framework that has been developed for children with disabilities. The comparable social inclusion outcomes of most of the young children with hearing loss to those with typical hearing is promising. This study adds to our knowledge of how social inclusion can be facilitated in young children with hearing loss and provides a methodology for measuring social inclusion that can be applied to other cohorts of children with disabilities. It also forms the foundation for future studies looking at factors influencing social inclusion for children with hearing loss.

## Abbreviations

LSAC: Longitudinal Study of Australian Children
LSL: Listening and spoken language
SDQ: Strength and difficulties questionnaire
SS: Standard scores
